# Tail-Risk-Aware Model Predictive Control for Hypoglycemia Mitigation in Automated Insulin Delivery

**DOI:** 10.3390/metabo16100759

**Published:** 2026-10-09

**Authors:** Kaiyuan Zhang, Congyu Han, Hexing Guo, Shuo Kang, Shuji Jing, Chunrui Wang, Tianyi Zang, Yanli Zhao

**Affiliations:** 1 Faculty of Computing, Harbin Institute of Technology, Harbin 150001, China; kaiyuan.zhang@hit.edu.cn (K.Z.); 25s003106@stu.hit.edu.cn (C.H.); 25s103357@stu.hit.edu.cn (H.G.); 25s103359@stu.hit.edu.cn (S.K.); 25s103248@stu.hit.edu.cn (S.J.); 25s103286@stu.hit.edu.cn (C.W.); 2 Medical College, Qinghai University, Xining 810016, China

**Keywords:** automated insulin delivery, type 1 diabetes, model predictive control, reinforcement learning, tail risk

## Abstract

**Background:** Automated insulin delivery must maintain glycemic control despite partial observability, physiological heterogeneity, and sensing and actuation errors. Cohort-average metrics may nevertheless conceal hypoglycemia in vulnerable virtual patients. **Methods:** We developed a tail-risk-aware model predictive control framework that combines a Transformer-based latent world model, unguided iterative cross-entropy method planning over perturbed rollouts, and a deterministic Safety Layer that projects insulin actions onto programmed bounds. Reinforcement-learning-guided proposal sampling was evaluated separately as an exploratory search variant. The primary controller was evaluated in Simglucose using 10 virtual patients, three random seeds, and five prespecified operating conditions. **Results:** Under nominal conditions, it achieved a cohort-average time in range (TIR) of 77.6% and time below range (TBR) of 3.8%, but worst-k TBR was 11.0% and increased to 37.4% under 5% random CGM missingness. In the risk-objective ablation, combining variance penalization with TailMean increased TIR by 2.8 percentage points and reduced cohort-average and worst-k TBR by 2.3 and 7.4 percentage points, respectively, relative to mean-only planning. The Safety Layer modified every proposed action in the analyzed run, indicating strong dependence on the programmed bounds. **Conclusions:** These results show that average target attainment can coexist with substantial patient-level risk. They support tail-aware evaluation and identify patient-specific safeguards as a priority for future validation, but do not establish clinical safety or efficacy.

## 1. Introduction

Automated insulin delivery (AID) systems integrate continuous glucose monitoring (CGM), insulin pumps, and control algorithms into a closed-loop framework for type 1 diabetes (T1D) management [1]. Unlike open-loop approaches that require manual insulin administration, AID systems adjust insulin delivery through real-time feedback control. A central objective is to maintain glucose within 70–180 mg/dL, commonly quantified as time in range (TIR), while limiting exposure below range [2,3,4,5]. Improved glycemic control may reduce immediate hypoglycemia burden and may contribute to management strategies intended to limit longer-term metabolic complications.

At its core, AID control represents a safety-critical sequential decision-making process where erroneous dosing decisions could trigger unacceptable hypoglycemic or hyperglycemic events. Consequently, control algorithms must simultaneously optimize primary efficacy endpoints such as TIR while rigorously managing risk metrics like Time-Below-Range (TBR) and hypoglycemia duration. Moreover, safety logic must remain auditable and traceable to satisfy regulatory requirements for medical device approval.

The landscape of AID controllers generally encompasses three major categories. Model-predictive control (MPC) leverages predictive models and explicit constraints for optimization, through which it gains advantages in interpretability and engineering deployment [6,7]. However, fixed or weakly adaptive models often struggle to capture the non-stationary dynamics and severe inter-individual variability inherent in T1D physiology, resulting in limited cross-subject robustness [8,9]. End-to-end policy learning, including both online and offline reinforcement learning (RL), has shown strong mean performance and scalability in controlled evaluations [10,11]. Nevertheless, the medical field cannot tolerate the risks associated with online exploration, and offline learning methods suffer from extrapolation errors and safety failures when policies deviate from the data support region [12]. Model-based planning approaches learn dynamics or latent world models from fixed datasets [13,14] and use those models for planning. Sampling-based MPC then optimizes objectives and constraints through model rollouts [15]. A key advantage of this approach is that risk preferences and programmed action constraints remain explicit rather than being absorbed entirely into black-box policy parameters.

This study adopts the model-based planning approach and emphasizes two design principles. First, a Transformer architecture [16] encodes historical CGM and insulin observations into latent representations for multi-step glucose prediction under partial observability. Second, an auditable Safety Layer maps proposed actions into programmed bounds through deterministic action projection and records each adjustment. This separation makes the relationship between planner proposals, action feasibility, and intervention magnitude traceable without treating the Safety Layer as a guarantee of future glucose safety [17]. The primary contributions of this work are as follows:

- A unified framework combining a Transformer-based latent world model with iCEM-MPC and stepwise action-feasibility auditing at each control cycle.
- An optional hybrid planning variant in which RL shapes the iCEM proposal distribution while the MPC objective retains authority over action selection; the unguided iCEM-MPC configuration remains the primary controller evaluated in this study.
- A Safety Layer audit that quantifies how often and how strongly planner proposals are modified by programmed action bounds.
- A risk-objective ablation that separates mean, variance, and TailMean terms, together with cohort-average and worst-k evaluation across five operating conditions.

## 2. Related Work and Study Rationale

This section organizes the research most relevant to the current study by algorithmic component. In automated insulin delivery (AID), model predictive control (MPC) is widely used because its objectives and constraints remain explicit [18]. It allows action bounds, maximum dosing rates, and prediction horizons to be encoded directly within the optimization problem. The UVA/Padova physiological model provides a standardized environment for evaluating controllers across virtual patients with diverse metabolic profiles [19,20]. The present study used its Simglucose implementation.

Regarding state estimation and glucose prediction, sequence models have demonstrated robust capacity to recover critical state summaries from noisy historical CGM data [21,22]. Specifically, attention mechanisms and recurrent architectures have been employed to encode multidimensional temporal histories into actionable decision representations, enabling the development of modular pipelines that link state estimation with downstream planning modules [23,24]. Recent advances in Transformer-based architectures have further enhanced the ability to capture long-range temporal dependencies in glucose dynamics. To prevent the coupling of predictors and controllers into uninterpretable black boxes, this work positions the sequence model as an auditable state estimation module through which risk preferences and physiological constraints are explicitly integrated into the control chain rather than being absorbed into opaque neural network parameters.

In the field of offline Reinforcement Learning (RL), policies are derived from fixed historical datasets to mitigate the ethical and safety risks associated with online exploration in clinical settings [25]. While deep RL methods for AID control have advanced rapidly in tasks such as automated bolus calculation and basal rate adjustment [26,27], distribution shift and policy extrapolation remain central challenges when learned actions depart from the support of the training data [12,25]. Recent AID studies have therefore examined hypoglycemia-focused control and structured basal–bolus decision making [28,29]. Reviews of RL-based glucose control also discuss risk-mitigation strategies such as constrained action spaces and execution-time constraint handling [30,31].

Furthermore, the world model paradigm has emerged as a promising approach that achieves flexible control by decoupling dynamics learning from planning [13,14]. This framework learns compact latent representations of the environment dynamics from offline data and utilizes sampling-based MPC to handle non-convex cost landscapes through parallelizable trajectory optimization. The Cross-Entropy Method (CEM) and its variants provide sampling-based tools for optimization in such settings [32,33]. This work employs an iterative CEM (iCEM) variant featuring time-correlated sampling and warm-start mechanisms initialized from previous solutions, as it offers an efficient approximate solution for non-convex optimization under finite computational budgets [34]. During execution, shielding and projection techniques can map planned outputs into prespecified feasible action sets [35,36]. These modules support constraint satisfaction in safety-critical control [37], but action feasibility alone does not guarantee a safe downstream state. Finally, this study uses worst-k analysis and other tail-risk measures to identify failures that cohort means can conceal.

## 3. Materials and Methods

The primary method combines a world model, robust iCEM-MPC, and an auditable Safety Layer in a tail-risk-aware online planner. The world model estimates state and predicts glucose, while iCEM-MPC performs risk-sensitive optimization through perturbed rollouts and tail aggregation. The Safety Layer enforces programmed action bounds through deterministic projection and logs intervention costs. The primary configuration uses unguided iCEM sampling. RL-guided proposal sampling is evaluated separately as an optional search variant and does not choose executed actions.

### 3.1. Preliminaries

Type 1 diabetes (T1D) control presents unique challenges that necessitate a Partially Observable Markov Decision Process (POMDP) formulation. The physiological dynamics governing blood glucose are highly nonlinear and exhibit significant inter-patient variability, making it impossible to directly observe the true underlying metabolic state. Continuous glucose monitoring (CGM) provides only a noisy, intermittently sampled projection of this latent state, with typical sampling intervals of 3 to 5 min introducing substantial measurement delays. Furthermore, subcutaneous insulin absorption follows a delayed pharmacokinetic profile, with peak action occurring approximately 60 min after administration. These compounding factors—delayed observations, lagged responses, and nonlinear physiology—motivate a POMDP formulation in which the controller must reason about hidden physiological states rather than relying on raw CGM readings alone.

We formalize the closed-loop insulin delivery task as

(1) $$M=\langle S,A,O,p\left(s_{t+1}|s_{t},a_{t}\right),p\left(o_{t}|s_{t}\right),r,\gamma \rangle ,$$

where $s_{t}\in S$ denotes the latent physiological state (e.g., plasma glucose concentration, insulin concentration, meal absorption rate), $o_{t}\in O$ is the CGM observation, $a_{t}\in A$ is the insulin dosing action, *r* is the control reward, and γ is the discount factor. The transition dynamics $p(s_{t+1}|s_{t},a_{t})$ capture the nonlinear glucose–insulin physiology, while the observation model $p(o_{t}|s_{t})$ models CGM noise and sampling artifacts.

To address partial observability, we construct parallel temporal histories at three scales, W={20,72,288}:

(2) $$h_{t}^{\left(w\right)}=\left(o_{t-w+1:t},a_{t-w+1:t-1}\right),\;w\in W,$$

where each history jointly encodes the most recent CGM readings and intervening insulin actions at the corresponding scale. The experimental sequences are aligned to the 5 min control cycle, so the three windows cover approximately 100 min, 6 h, and 24 h. The short branch covers the immediate glucose–insulin response, the intermediate branch represents within-day trends, and the long branch provides context for recurring daily and circadian patterns. The collection $H_{t}=\left\{h_{t}^{\left(20\right)},h_{t}^{\left(72\right)},h_{t}^{\left(288\right)}\right\}$ serves as the input to the downstream controller at time *t*.

Formally, the underlying Markov Decision Process (MDP) of the closed-loop insulin delivery system is defined by the tuple 〈S,A,p,r,γ〉. The state space S encompasses all relevant physiological variables, including plasma glucose concentration g∈[50,400] mg/dL, plasma insulin concentration $i\in R_{\geq 0}$, and meal absorption rate $m\in R_{\geq 0}$. The action space A⊂R represents the insulin dosing decisions, constrained to $a_{t}\in \left[0,a_{\max}\right]$ with $a_{\max}=10$ units per step. The transition dynamics $p(s_{t+1}|s_{t},a_{t}):S\times A\to S$ encode the nonlinear glucose-insulin pharmacokinetic and pharmacodynamic response, modeled via the UVA/Padova type 1 diabetes simulator. The reward function $r(s_{t},a_{t})$ is designed to align with clinical glycemic targets:

(3) $$\begin{array}{c} r\left(s_{t},a_{t}\right)=w_{1}I\left[g_{t}\in \left[70,180\right]\right]-w_{2}I\left[g_{t}<70\right]-w_{3}I\left[g_{t}>250\right]-c\cdot a_{t}, \end{array}$$

where $g_{t}$ is plasma glucose at control step *t*, $w_{1}$, $w_{2}$, and $w_{3}$ are clinical priority weights, I[·] is the indicator function, and *c* penalizes excessive insulin usage. The discount factor γ∈(0,1) balances immediate and long-term glycemic outcomes. A stochastic policy $\pi (a_{t}|z_{t})$ maps the latent state estimate to an insulin dosing distribution, with the objective of maximizing the expected cumulative return $J\left(\pi \right)=E_{\pi }\left[\sum_{t=0}^{T}\gamma^{t}r\left(s_{t},a_{t}\right)\right]$. The state-value function $V^{\pi }\left(z\right)=E_{\pi }\left[\sum_{t=0}^{\infty }\gamma^{t}r\left(s_{t},a_{t}\right)|s_{0}=z\right]$ and action-value function $Q^{\pi }\left(z,a\right)=E_{\pi }\left[\sum_{t=0}^{\infty }\gamma^{t}r\left(s_{t},a_{t}\right)|s_{0}=z,a_{0}=a\right]$ follow the standard Bellman equations, as summarized in Table 1.

**Figure 1:**
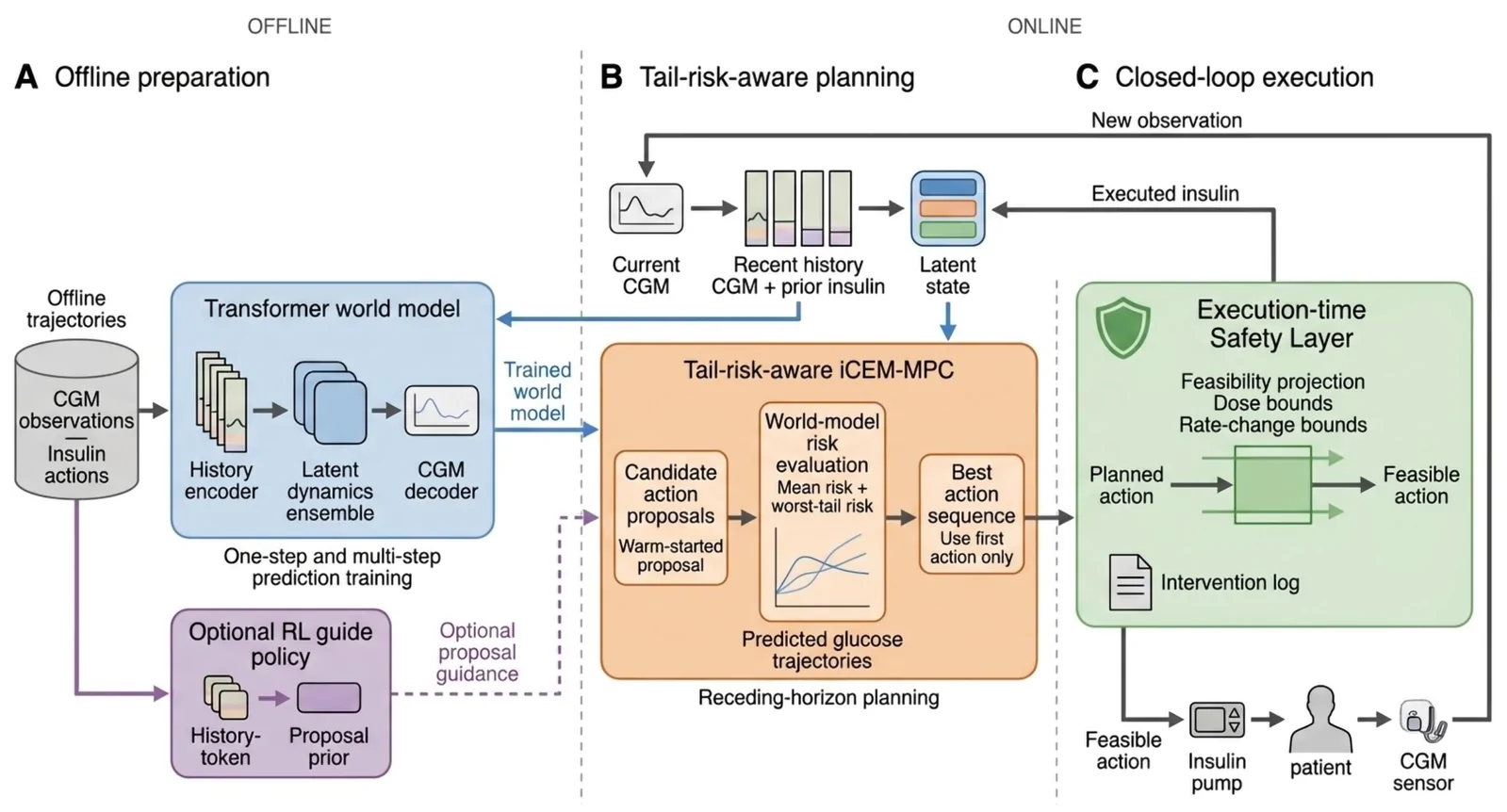
Architecture of the tail-risk-aware automated insulin delivery framework.

Figure 1 illustrates the overall framework, which consists of three core modules: the Transformer world model for state estimation, the iCEM-MPC planner for risk-sensitive action optimization, and the Safety Layer for execution-time action-feasibility enforcement. The guide-policy path shown in the figure is optional; setting the guidance ratio to zero yields the primary unguided controller.

### 3.2. Transformer Based Latent Variable World Model

The world model learns a compact latent representation of the T1D dynamics purely from offline trajectory data. It consists of three cascaded modules: a Transformer-based state encoder, a latent dynamics model, and an observation decoder. This design decouples state estimation from planning, ensuring that each module can be trained, audited, and updated independently.

**Multi-scale state encoder.** The encoder $e_{\theta }$ maps the parallel history set $H_{t}$ into a fixed-dimensional latent state $z_{t}\in R^{d}$. At each scale w∈{20,72,288}, CGM observations and insulin actions are represented as paired tokens with positional encodings and processed by a parallel Transformer branch. Each branch comprises *L* Transformer layers with multi-head self-attention (each head operates on dimension $d_{\mathrm{head}}=d/n_{\mathrm{heads}}$), layer normalization, and position-wise feedforward layers, producing a scale-specific representation $z_{t}^{\left(w\right)}$. A learned gating function assigns normalized weights to the scale-specific representations and fuses them:

(4) $$\alpha_{t}^{\left(w\right)}=\frac{\exp\;\left(q_{\phi }\left(z_{t}^{\left(w\right)}\right)\right)}{\sum_{v\in W}\exp\;\left(q_{\phi }\left(z_{t}^{\left(v\right)}\right)\right)},\;z_{t}=\sum_{w\in W}\alpha_{t}^{\left(w\right)}z_{t}^{\left(w\right)}.$$

The short, intermediate, and 24 h branches allow the encoder to attend jointly to recent glucose–insulin dynamics, within-day trends, and recurring daily context. The fused representation summarizes the estimated metabolic state for the downstream latent dynamics model and controller.

**Latent dynamics model.** The transition model $f_{\theta }\left(z_{t},a_{t}\right)\to {\hat{z}}_{t+1}$ is implemented as an MLP that predicts the next latent state given the current latent state and the planned action. We choose an MLP rather than an autoregressive RNN because the MPC planner requires repeated forward rollouts per decision step. The MLP supports batched candidate evaluation, whereas recurrent unrolling would add sequential operations. The latent dimensionality *d* balances model capacity against overfitting on the offline dataset.

**Observation decoder.** The decoder $d_{\theta }\left({\hat{z}}_{t+1}\right)\to {\hat{o}}_{t+1}$ maps the predicted latent state back to CGM space for reconstruction-based training and uncertainty quantification. Together, the three modules form the forward pass:

(5) $$z_{t}=e_{\theta }\left(h_{t}\right),\;{\hat{z}}_{t+1}=f_{\theta }\left(z_{t},a_{t}\right),\;{\hat{o}}_{t+1}=d_{\theta }\left({\hat{z}}_{t+1}\right).$$

**Training objective.** The world model is trained to minimize the prediction error between predicted and actual CGM observations. The primary loss is the one-step prediction error, which provides the strongest gradient for representation learning. To prevent error accumulation during multi-step planning rollouts, we additionally include a multi-step rollout loss:

(6) $$L_{\mathrm{pred}}=E\left[{∥o_{t+1}-{\hat{o}}_{t+1}∥}_{2}^{2}\right]+\sum_{k=1}^{K}\lambda_{k}\;E\left[{∥o_{t+k}-{\hat{o}}_{t+k}∥}_{2}^{2}\right],$$

where $\lambda_{k}$ controls the contribution of longer rollout steps and *K* is the maximum rollout horizon during training. This formulation encourages the latent dynamics model to learn a representation that remains accurate across multiple prediction steps, directly supporting the MPC planning use case.

**Dynamics ensemble for epistemic uncertainty.** To enable risk-sensitive planning, we train an ensemble of *M* independently initialized world models. Each ensemble member produces its own CGM prediction ${\hat{o}}_{t}^{\left(m\right)}$, from which we compute the ensemble mean and variance:

(7) $$\mu_{t}=\frac{1}{M}\sum_{m=1}^{M}{\hat{o}}_{t}^{\left(m\right)},\;\sigma_{t}^{2}=\frac{1}{M}\sum_{m=1}^{M}{\left({\hat{o}}_{t}^{\left(m\right)}-\mu_{t}\right)}^{2}.$$

The mean $\mu_{t}$ serves as the point prediction, while the ensemble variance $\sigma_{t}^{2}$ is used as a proxy for epistemic uncertainty. Because ensemble disagreement is not itself a calibrated uncertainty estimate, it is interpreted only as a relative signal of model disagreement. This signal enters the risk-sensitive MPC objective and penalizes candidate trajectories in regions with greater disagreement.

### 3.3. Model Predictive Control Objective Function

At each control step, the MPC planner optimizes an action sequence over a receding planning horizon. Given the current latent state $z_{t}$ estimated by the world model, MPC solves:

(8) $$a_{t:t+H-1}^{*}=\arg\min_{a_{t:t+H-1}}\;J\left(a_{t:t+H-1}\right),$$

where $a_{t:t+H-1}=\left(a_{t},a_{t+1},\ldots ,a_{t+H-1}\right)$ is the candidate action sequence, *H* is the planning horizon (measured in control steps), and *J* is the total cost over the horizon. After solving, only the first action $a_{t}^{*}$ is executed; at the next step the problem is re-solved with updated state information, implementing the standard receding horizon paradigm.

**Stage cost.** The cost at each step comprises four terms that together encode clinical priorities:

(9) $$\ell_{t}=\alpha_{\ell }{\left[70-{\hat{g}}_{t}\right]}_{+}^{2}+\alpha_{h}{\left[{\hat{g}}_{t}-180\right]}_{+}^{2}+\alpha_{a}{∥a_{t}∥}_{2}^{2}+\alpha_{\Delta a}{∥a_{t}-a_{t-1}∥}_{2}^{2}.$$

The first two terms penalize predicted hypoglycemia (${\hat{g}}_{t}<70$ mg/dL) and hyperglycemia (${\hat{g}}_{t}>180$ mg/dL) using weighted squared-hinge losses, where ${\left[x\right]}_{+}=\max\left(x,0\right)$. The quadratic penalties increase with the magnitude of a predicted violation and are compatible with the population-based planner used below. The hyperparameters $\alpha_{\ell }$ and $\alpha_{h}$ encode the relative weighting of low and high glucose, with a larger weight assigned to predicted hypoglycemia. The third term penalizes large insulin doses, and the fourth term penalizes rapid changes in dosing rate. These are algorithmic penalties rather than clinical guarantees.

**Risk-sensitive planning.** The baseline MPC cost *J* optimizes expected performance, but worst-case perturbations can drive dangerous hypoglycemia in tail patients. To account for model uncertainty, we augment the cost with a variance penalty:

(10) $$J_{\mathrm{risk}}=E\left[\ell_{t}\right]+\beta \;\mathrm{Var}\left({\hat{g}}_{t:t+H}\right),$$

where β≥0 controls conservativeness. A larger β penalizes high-variance glucose predictions, pushing the planner toward actions that are robust across the ensemble of world models. This provides a tunable knob between aggressive mean-optimizing behavior and conservative worst-case hedging.

**Tail-aggregated robustness.** While variance penalization handles epistemic uncertainty within the world model, we additionally ensure robustness to execution-time perturbations (sensor noise, action noise, dropout) through Monte Carlo sampling. At each planning step, we sample $N_{\mathrm{pert}}$ perturbed execution scenarios and roll out the candidate action sequence through the world model under each perturbation. These rollouts yield a distribution of glucose trajectories. Rather than taking the mean cost (which can mask dangerous tail outcomes), we apply the TailMean operator:

(11) $${\mathrm{TailMean}}_{\rho }\left({\left\{c_{j}\right\}}_{j=1}^{N}\right)=\frac{1}{|I_{\rho }|}\sum_{j\in I_{\rho }}c_{j},\;I_{\rho }={\mathrm{TopK}}_{\rho }\left(\left\{c_{j}\right\}\right),$$

which selects the largest ρ fraction of empirical costs (e.g., ρ=0.2 for the worst 20%) and averages them. This empirical upper-tail objective gives adverse rollout trajectories greater influence than an arithmetic mean. It is closely related to a sample-based conditional value-at-risk estimate; no differentiability claim is required because iCEM uses ranked population evaluations rather than gradient descent.

### 3.4. Cross-Entropy Method for Continuous Planning

The MPC objective is non-convex due to the nonlinear world model and the min-over-horizon structure, making gradient-based optimization unreliable. We therefore employ the Cross-Entropy Method (CEM), adapted as iCEM, as the continuous action planner. iCEM is a population-based evolutionary optimizer that maintains a Gaussian distribution over action sequences and iteratively refines it using elite samples from the previous iteration.

At iteration *k*, candidate action sequences are sampled from the current Gaussian proposal:

(12) $$u_{i}^{\left(k\right)}∼N\left(\mu^{\left(k\right)},\Sigma^{\left(k\right)}\right),\;i=1,\ldots ,N,$$

where *N* is the population size, $\mu^{\left(k\right)}$ is the mean action sequence, and $\Sigma^{\left(k\right)}$ is the covariance matrix. After evaluating all *N* candidates through the world model rollouts and computing their costs, we select the top fraction (e.g., the top 10%) as elite samples and update the Gaussian parameters using elite statistics:

(13) $$\mu^{\left(k+1\right)}=\left(1-\eta \right)\mu^{\left(k\right)}+\eta \;{\hat{\mu }}_{\mathrm{elite}}^{\left(k\right)},\;\Sigma^{\left(k+1\right)}=\left(1-\eta \right)\Sigma^{\left(k\right)}+\eta \;{\hat{\Sigma }}_{\mathrm{elite}}^{\left(k\right)}.$$

The exponential moving average (EMA) with coefficient η prevents abrupt distribution shifts between iterations, promoting stable convergence. The full optimization loop runs for *K* iterations (e.g., K=8), progressively concentrating the search distribution around low-cost regions.

**Warm-start from receding horizon.** To reduce computational requirements and enhance closed-loop stability, we initialize each planning problem using the solution from the previous time step. Specifically, the mean at the first iteration is set to the shifted optimal sequence from the previous control cycle:

(14) $$\mu^{\left(0\right)}\leftarrow \left(a_{t+1}^{*},a_{t+2}^{*},\ldots ,a_{t+H-1}^{*},a_{t+H-1}^{*}\right),$$

where the last action is repeated to maintain sequence length. This warm-start strategy exploits temporal coherence between successive planning problems, dramatically reducing the number of iterations needed for convergence and ensuring that the controller’s behavior is smooth across time steps.

To prevent the proposal distribution from becoming stale after an abrupt disturbance, the warm start is reset when the observed CGM rate of change exceeds 3 mg/dL/min for two consecutive control cycles or when ensemble disagreement exceeds the 95th percentile estimated from the training set. Following a reset, 50% of candidates are drawn from a broad diagonal proposal with σ=0.50 U, while the remaining 50% are drawn from the shifted warm-start distribution. This event-triggered mechanism was evaluated under the prespecified disturbance conditions and does not guarantee recovery from arbitrary disturbances.

**iCEM implementation.** The implementation augments the standard CEM update with warm starts and temporally correlated sampling. Candidate sequences are ranked by their evaluated costs, and the proposal distribution is updated from the elite subset. These choices reduce the number of model evaluations required within the fixed planning budget, but they do not guarantee a global optimum.

### 3.5. Execution-Time Safety Layer

World-model predictions remain imperfect, and proposed actions may fall outside the programmed dosing bounds. We therefore apply a deterministic Safety Layer after planning and during model rollouts. The layer is implemented as projection onto a programmed feasible action set.

The Safety Layer defines a feasible action set $A_{\mathrm{feas}}$ that encodes programmed bounds on insulin dosing:

(15) $$A_{\mathrm{feas}}=\left\{a\in R:0\leq a\leq a_{\max},\;\left|a-a_{t-1}\right|\leq \Delta a_{\max}\right\},$$

where $a_{\max}$ is the maximum insulin dose per control cycle and $\Delta a_{\max}$ is the maximum rate of dose change. All virtual patients were evaluated under the same programmed bounds. Projection onto this set enforces the dose and rate-of-change constraints but does not guarantee a safe future glucose trajectory.

At each control step, the Safety Layer receives the MPC-proposed action $a_{t}^{\mathrm{plan}}$ and projects it onto the feasible set:

(16) $${\tilde{a}}_{t}=\arg\min_{a\in A_{\mathrm{feas}}}{∥a-a_{t}^{\mathrm{plan}}∥}_{2}^{2}.$$

This projection can be solved analytically as a box constraint with an additional linear bound, and the solution is unique for the stated convex set. The projected action is the closest feasible action to the MPC proposal in Euclidean distance. This property guarantees feasibility with respect to the programmed bounds, not a safe glucose outcome.

**Projection audit metrics.** To characterize the layer’s effect, we maintain three audit metrics:

(17) $$I_{t}=1\left[{\tilde{a}}_{t}\neq a_{t}^{\mathrm{plan}}\right],\;D_{I}=\sum_{t=1}^{T}I_{t},\;I_{1}=\frac{1}{T}\sum_{t=1}^{T}{∥a_{t}-{\tilde{a}}_{t}∥}_{1}.$$

$I_{t}$ is a binary indicator of whether intervention occurred at step *t*. $D_{I}$ measures the total number of modified actions. A high value may indicate aggressive MPC proposals, restrictive action bounds, or both. $I_{1}$ is the mean L1 adjustment per control step, consistent with the denominator in Equation (10). These metrics quantify algorithmic intervention and do not by themselves establish clinical safety.

The Safety Layer uses a deterministic rule so that every modification can be reproduced from the proposed action and programmed bounds. It also avoids introducing a second learned component into the action-feasibility check.

### 3.6. Rollout and Distribution Alignment

A planning–execution mismatch arises when the Safety Layer modifies actions after they have been evaluated. If model rollouts use unprojected candidates but the environment executes projected actions ${\tilde{a}}_{t}$, predicted trajectories no longer correspond to the executed action sequence.

This mismatch affects tail-risk estimation because the planner evaluates one action sequence while glucose evolves under another. Candidate costs can therefore be systematically misestimated when projection is omitted from model rollouts.

To align planning and execution, the same Safety Layer mapping is applied within MPC rollouts. Each candidate generated by iCEM is projected before advancing the world model:

(18) $$z_{t+h+1}=g_{\phi }\left(z_{t+h},\;{\tilde{a}}_{t+h}\right),\;{\tilde{a}}_{t+h}=\Pi_{A_{\mathrm{feas}}}\left(a_{t+h}^{\mathrm{plan}}\right),\;{\hat{o}}_{t+h+1}=d_{\psi }\left(z_{t+h+1}\right).$$

The projected action ${\tilde{a}}_{t+h}$ is used for both world-model rollout and cost evaluation. This removes the avoidable action mismatch between the optimization path and the executed control path.

### 3.7. Optional RL-Guided Proposal Sampling

We additionally evaluate a hybrid search variant in which RL serves as a proposal generator while MPC retains authority over the planning objective. This variant is not the primary controller used for the main cross-method and cross-domain results. RL contributes learned proposal priors, and the Safety Layer applies the same programmed bounds during rollout and execution. RL is confined to constructing candidate distributions and never directly outputs executed actions.

**Guided sampling.** Let $\pi_{g}$ denote the guide policy, which is trained via offline RL on the Simglucose dataset. Unlike the MPC planner, which starts each planning problem from scratch, $\pi_{g}$ encodes learned insulin dosing priors into a structured proposal. At iteration *k* of iCEM, candidate action sequences are sampled from a mixture distribution:

(19) $$q^{\left(k\right)}=\left(1-\lambda \right)\;N\left(\mu^{\left(k\right)},\Sigma^{\left(k\right)}\right)+\lambda \;N\left(\mu_{g},\sigma_{g}^{2}\Sigma^{\left(k\right)}\right),$$

where λ∈[0,1] controls the injection strength of the guide prior. When λ=0, the method reduces to vanilla iCEM; when λ=1, it reduces to pure guide-policy sampling. The guide noise scale $\sigma_{g}$ controls how far candidate samples deviate from the guide’s mean action, balancing exploitation of learned priors against exploration of novel action sequences. A small fraction of the iCEM population is also reserved as a deterministic candidate equal to the current mean trajectory for stability.

**Receding-horizon guide rollout.** A single-step action is insufficient to capture the long-term glucose dynamics, because insulin pharmacokinetics involve a delayed peak action approximately 60 min after administration. We therefore generate an *H*-step guide trajectory using the world model for recursive dynamics prediction. Starting from the current latent state $z_{t}$, the guide policy proposes the first action, the world model transitions to the next latent state, and the process repeats recursively:

(20) $$z_{t+h+1}=g_{\phi }\left(z_{t+h},\;\Pi_{A_{\mathrm{feas}}}\left(\pi_{g}\left(h_{t+h}\right)\right)\right),\;h=0,\ldots ,H-1.$$

The projected action $\Pi_{A_{\mathrm{feas}}}\left(\pi_{g}\left(\cdot \right)\right)$ is used at each step to respect the programmed bounds during rollout. This yields an *H*-step guidance sequence $\left({\hat{a}}_{t},{\hat{a}}_{t+1},\ldots ,{\hat{a}}_{t+H-1}\right)$ that is subsequently used as the guide prior $\mu_{g}$ for the iCEM sampling distribution. The recursive rollout captures delayed insulin effects across the planning horizon.

**Perturbed guide rollout.** To represent action-noise sensitivity during guidance, we sample *J* perturbed versions of the guide action at each rollout step:

(21) $$a_{t+h}^{\left(j\right)}=\Pi_{A_{\mathrm{feas}}}\left(a_{t+h}^{g}+\epsilon_{h}^{\left(j\right)}\right),\;\epsilon_{h}^{\left(j\right)}∼N\left(0,\sigma_{\epsilon }^{2}I\right),\;j=1,\ldots ,J.$$

Each perturbed action is rolled out through the world model, producing *J* glucose trajectory samples. Rather than averaging these (which would mask dangerous outliers), we aggregate them using a hypoglycemia-oriented TailMean: the worst ρ fraction of the *J* glucose values is selected and averaged. This tail-aggregated prediction updates the predictive history $h_{t+h}$ for the next guide step. The effect is that the guide policy proactively favors conservative action branches during planning, improving robustness against action noise for the most vulnerable tail patients.

### 3.8. Evaluation Protocol

The evaluation framework used time in range (TIR) as the primary endpoint and time below range (TBR), coefficient of variation (CV), worst-k performance, and Safety Layer intervention as complementary outcomes. Table 2 summarizes the definitions used in the analyses. Clinical targets are provided as interpretive reference points for cohort-average outcomes; meeting these targets alone does not establish clinical safety or efficacy.

To prevent aggregate means from obscuring heterogeneous outcomes, metrics were summarized as both cohort averages and worst-k values. For TIR, the worst-k set contained the lowest 20% of virtual-patient values; for TBR, it contained the highest 20%. Worst-k is therefore a descriptive subgroup summary rather than a formal worst-case guarantee.

Robustness was evaluated under five operating conditions: nominal operation and four perturbations comprising action noise (action_noise_0p5), CGM noise (cgm_noise_10), CGM dropout (cgm_dropout_0p05), and action delay (action_delay_1). Action noise had Gaussian variance $\sigma^{2}=0.5$, CGM noise had σ=10 mg/dL, CGM dropout had 5% missingness, and action delay was one control cycle. These controlled stress tests do not represent the full range of real-world device failures.

For the unannounced-meal stress test, a carbohydrate meal was introduced at a prespecified time while its timing and size were withheld from the controller. Challenges of 30 g, 60 g, and 90 g were compared with the nominal condition using TIR, TBR, time below 54 mg/dL, worst-k TBR, peak glucose, and time to return below 180 mg/dL. This scenario characterizes the limits of inferring meal effects indirectly from CGM and insulin history rather than robustness to unrestricted meal disturbances.

### 3.9. Data Sources and Experimental Settings

The primary controller integrates a Transformer world model with tail-risk-aware iCEM-MPC and the Safety Layer and uses unguided iCEM proposal sampling. An optional hybrid variant adds RL-guided proposal sampling while retaining the same MPC objective and Safety Layer. End-to-end PPO, SAC, and TD3 policies, each coupled to the same Safety Layer, serve as comparative baselines.

The contribution of the risk objective was evaluated using four planner configurations: mean cost only, mean cost with the ensemble-variance penalty, mean cost with TailMean aggregation, and the complete objective containing both the variance penalty and TailMean. Outcomes were evaluated in the nominal domain and the four perturbation domains defined above. Because no uncertainty intervals or hypothesis tests were available for this ablation, the comparisons were treated as descriptive.

The training dataset combined records from MIMIC-IV [38] with Simglucose trajectories to yield 83,600 transitions. MIMIC-IV records were selected for patients with an ICD-10 diagnosis in the E10.* category and required the availability of both glucose and insulin records. MIMIC-IV was used only as an auxiliary source for representation learning. Its intensive-care setting, patient acuity, sampling density, treatment context, insulin administration, and comorbidity profile are not equivalent to outpatient type 1 diabetes management, and these records were not used as clinical validation of the controller. Table 3 reports the data composition available for this study.

To assess source shift, otherwise-matched world models were trained using Simglucose alone, MIMIC-IV alone, and the combined sources. Source separability was quantified with a held-out source classifier and latent-representation maximum mean discrepancy. Prediction error was evaluated on held-out Simglucose trajectories, and closed-loop evaluation was restricted to models with an input–action interface compatible with Simglucose. This analysis tests whether adding the auxiliary ICU source degraded performance in the target evaluation domain; it does not establish outpatient or clinical transfer.

### 3.10. Reproducibility and Statistical Reporting

The controller operated on a 5 min control cycle. The reported planner configuration used a horizon of H=12, a population of N=64 candidate sequences, and 768 forward predictions per decision step. The primary benchmark experiments used 10 virtual patients and three random seeds, comprising 64,296 evaluation minutes; a separate sensitivity analysis used an expanded cohort of 30 virtual patients. Each primary-benchmark virtual-patient–seed combination contributed one observation per method to the pairwise summaries (n=30 observations per method). The same virtual patient under the same random seed was evaluated with the primary unguided iCEM-MPC controller, PPO, SAC, and TD3.

Table 4 summarizes the experimental configuration. The primary cohort comprised a prespecified benchmark subset of 10 adult and adolescent Simglucose subjects with complete 24 h meal schedules and no missing model parameters. This subset supports reproducible comparison but is not representative of a clinical population. Sensitivity analyses included 30 virtual subjects (12 adults, 10 adolescents, and 8 children), each evaluated with three predefined random seeds.

For each baseline comparison, outcomes were matched by virtual patient and random seed. Paired differences were calculated as the proposed-method outcome minus the corresponding comparator outcome. The analysis unit was one patient–seed pair (n=30). Formal normality and variance-homogeneity tests were not used as prerequisites for selecting the inferential procedure. Because the sample was small and Gaussian outcome distributions could not be assumed, the analysis used 10,000-resample bootstrap intervals and two-sided Wilcoxon signed-rank tests. Holm adjustment was applied across comparisons. These analyses were exploratory rather than confirmatory and were not part of a prespecified clinical-efficacy testing strategy. Outcomes were available for every method and matched combination; no runs were excluded and no values were imputed.

## 4. Results

### 4.1. Main Results

To validate the effectiveness of the proposed method in addressing the challenging trade-off between therapeutic efficacy and risk, this section designs a series of comparative experiments focused on dynamic control. These include comparative analyses of different control strategies, an assessment of the reinforcement learning guidance mechanism’s effectiveness, and an evaluation of system robustness under various perturbation domains.

**Figure 2:**
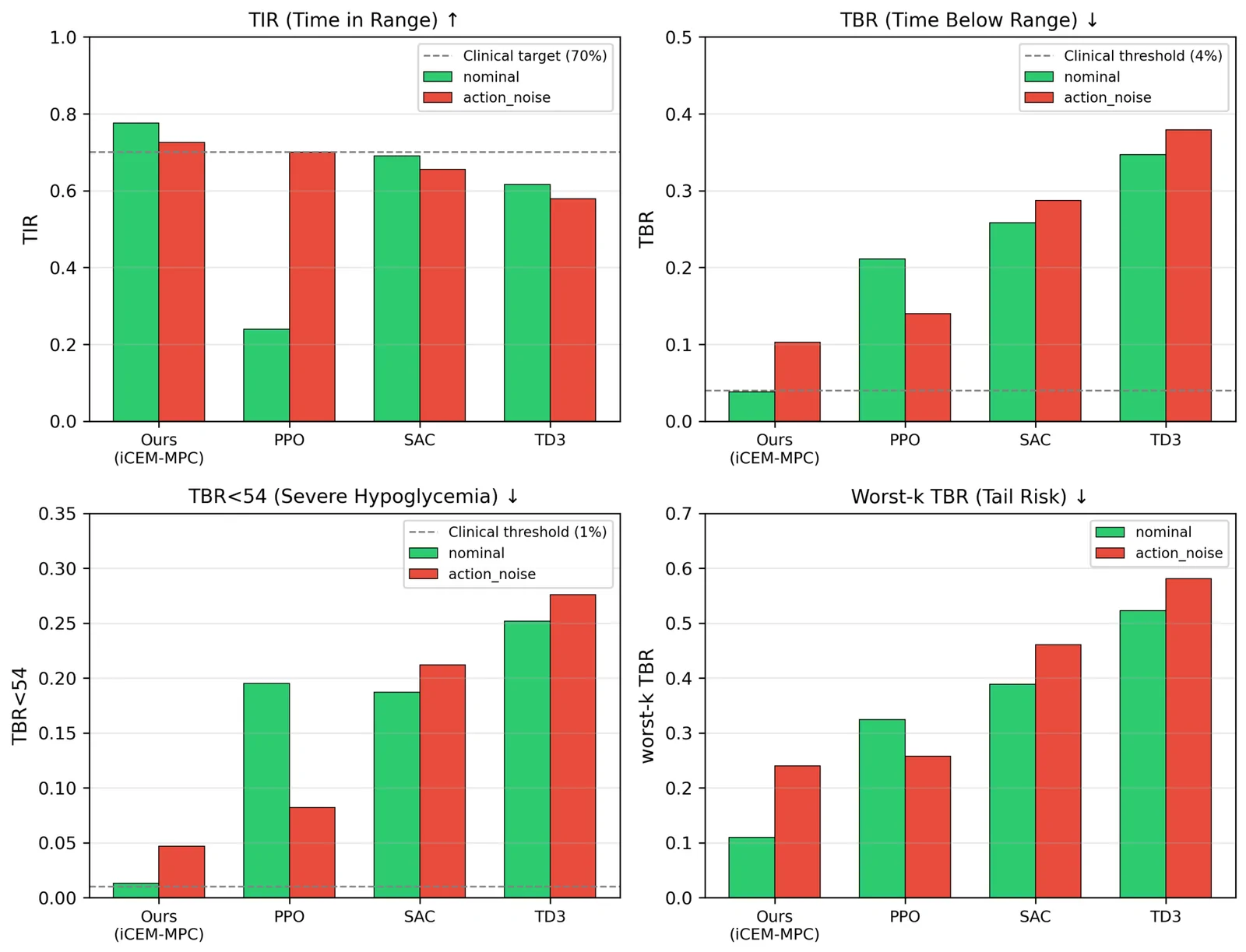
TIR, TBR, time below 54 mg/dL, and worst-k TBR for the proposed controller and reinforcement-learning baselines under nominal and action-noise conditions. Bars in the TIR, TBR, and time-below-54 panels show cohort means; bars in the worst-k TBR panel show the corresponding worst-k subgroup summaries. An upward arrow indicates that a higher value is better, while a downward arrow indicates the opposite.

Figure 2 compares the control strategies. Under nominal conditions, the proposed method achieved a cohort-average Time-in-Range (TIR) of 77.6% and Time-below-Range (TBR) of 3.8%. Under action noise, TIR decreased to 72.6% and TBR increased to 10.3%. These stress-test outcomes do not establish robust or clinically acceptable control.

PPO achieved a cohort-average TIR of 23.9%, while SAC and TD3 yielded cohort-average TBR values of 25.8% and 34.7%, respectively. These values represent implementation-level comparisons under the matched evaluation protocol. The TBR of the proposed method was 0.038, approximately 11% of the TD3 value of 0.347. A worst-k comparison cannot be made without the corresponding TD3 worst-k estimate.

These contrasts do not demonstrate clinical efficacy or isolate the reduction in hypoglycemia from differences in insulin delivery. The cohort-average and worst-k estimates are descriptive and depend on the evaluation protocol, common action constraints, and Safety Layer intervention pattern.

In Table 5, the confidence intervals excluded zero for all exploratory contrasts except SAC TIR. The mean SAC TIR difference was 0.017 (95% CI, −0.056 to 0.090; Holm-adjusted p=0.999), whereas the SAC TBR difference was −0.101 (95% CI, −0.177 to −0.025; Holm-adjusted p=0.048). These estimates apply to the evaluated protocol.

### 4.2. Exploratory RL-Guidance Study

**Figure 3:**
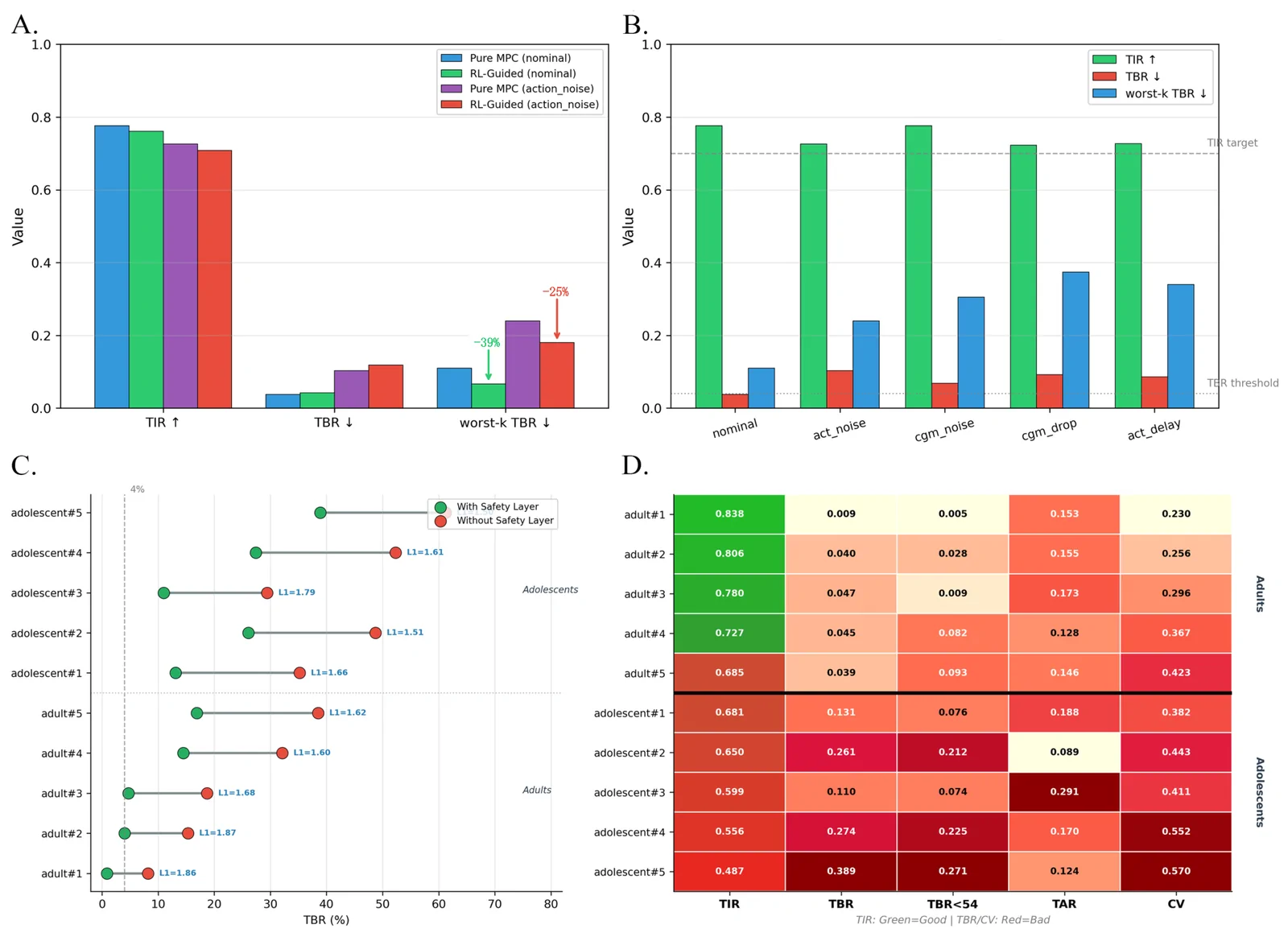
(**A**) Effect of RL-guided proposal sampling; (**B**) performance across domains; (**C**) Safety Layer interventions; and (**D**) patient outcomes. Black arrows: pointing up means a higher value is better, and vice versa; green/red arrows: indicate a decrease in the corresponding value.

The effectiveness of the reinforcement learning (RL)-guided mechanism is illustrated in Figure 3A; the RL-guided MPC underperforms traditional MPC on certain aggregate control metrics. In the nominal scenario, the TIR for RL-guided MPC is 0.76 (lower than the 0.775 achieved by traditional MPC), while the TBR increases from 0.038 to 0.04; under action noise conditions, the TIR drops from 0.725 to 0.707, and the TBR rises from 0.108 to 0.120. These results indicate that, under identical experimental conditions, the RL strategy does not yield a significant improvement in average blood glucose control and even incurs some performance loss.

The primary reason for this is that the RL strategy’s optimization objective extends beyond TIR and average TBR; the reward function places significant weight on penalizing severe hypoglycemia and sustained risk. Consequently, to mitigate extreme hypoglycemic events, the system may adopt a more conservative control strategy—such as limiting fluctuations in control inputs or preemptively reducing insulin infusion rates. While this enhances safety, it can slow the return of blood glucose levels to the target range, causing levels to remain outside the target zone for longer periods, ultimately manifesting as decreased TIR and increased TBR. Furthermore, whereas traditional MPC utilizes a system model for online receding-horizon prediction and recalculates control inputs in real-time based on current glucose status, the RL strategy relies primarily on state-action mappings learned during training. In the presence of action noise, it is susceptible to issues such as insufficient training sample coverage and policy approximation errors, resulting in relatively weaker real-time corrective capabilities.

It should be noted, however, that the RL-guided MPC does not underperform traditional MPC across all metrics. Specifically, the “worst-k” TBR—representing performance during the most adverse periods—decreases from 0.11 to 0.067 in the nominal scenario and from 0.24 to 0.18 under action noise conditions, representing reductions of approximately 39% and 25%, respectively. This demonstrates that, while the RL approach sacrifices some average control performance, it significantly reduces the risk of hypoglycemia during the most critical time intervals.

### 4.3. Performance Across Operating Conditions

Figure 3B summarizes the primary unguided iCEM-MPC controller under nominal operation and four controlled perturbations. The stress tests probe sensitivity to sensing and actuation errors but do not reproduce the full distribution of real-world device failures.

**CGM noise (****cgm_noise_10****).** With Gaussian sensor noise of σ=10 mg/dL, cohort-average TIR remained 0.776, whereas worst-k TBR increased to 0.305. Thus, a stable cohort average coexisted with substantial degradation in the highest-TBR subgroup.

**Action noise (action_noise_0p5).** Action noise reduced cohort-average TIR from 0.776 to 0.726 and increased cohort-average TBR from 0.038 to 0.103. Worst-k TBR reached 0.240.

**CGM dropout (cgm_dropout_0p05).** With 5% of CGM readings missing at random, cohort-average TIR was 0.723 and TBR was 0.092. Worst-k TBR reached 0.374, the highest value among the evaluated domains. This is a random-missingness stress test, not a continuous sensor-failure scenario.

**Action delay (action_delay_1).** A one-cycle action delay produced a cohort-average TIR of 0.727 and TBR of 0.086. Worst-k TBR increased to 0.340.

Across the four perturbations, cohort-average TIR differed from nominal by at most 5.3 percentage points. In contrast, worst-k TBR ranged from 0.240 to 0.374. The divergence between cohort-average and subgroup outcomes motivated the separate tail-risk analysis below.

### 4.4. Safety Layer Interventions and Patient Outcomes

Because insulin sensitivity differs across age groups, the Safety Layer intervention pattern was evaluated separately from downstream glucose safety. The intervention rate was 100% in the analyzed run, meaning that every proposed action was modified before execution. This pattern may arise from restrictive programmed bounds, aggressive or poorly calibrated planner proposals, or both.

The mean absolute adjustment was 1.665 insulin units per control step. Because the full intervention distribution was unavailable, the magnitude of this adjustment cannot be interpreted as minor fine-tuning. Projection enforced the programmed bounds but did not establish safe future glucose trajectories. The age-group results are descriptive subgroup findings, and the individual TBR range of 27–39% is not directly comparable with the subgroup mean from the expanded cohort.

We conducted a detailed analysis across age groups to gain deeper insight into the method’s performance for different populations, focusing on the physiological parameters and glycemic stability of adults versus adolescents. As illustrated in Figure 3D, adult patients achieved stable glycemic control, with TBR consistently below 5%. Conversely, adolescent patients faced significant clinical challenges regarding glycemic control, evidenced by markedly elevated TBR values. This difference stems from the fact that insulin sensitivity in adolescents is approximately double that of the group with stable blood glucose levels. Additionally, the coefficient of variation (CV) was closely correlated with TBR; specifically, blood glucose fluctuations were pronounced when the CV exceeded 0.36. This pattern indicates severe and unpredictable glycemic volatility, and high glycemic variability is a primary predictor of hypoglycemia risk. From the perspective of personalized therapy, these findings suggest that risk stratification should be implemented during system deployment: for patients exhibiting high sensitivity and high glycemic variability, more conservative initial parameters, stricter safety thresholds, and more frequent online calibration are required; conversely, for stable patients, control sensitivity can be increased to improve Time in Range (TIR). In other words, a uniform set of control parameters cannot adequately serve the entire patient population, and stratification based on patient age is a critical step in translating algorithmic performance into clinical benefits.

Furthermore, Safety-Layer statistics and analyses of patient-specific differences can provide complementary insights; if a specific patient subgroup consistently triggers frequent control adjustments, it often indicates that the upstream model or cost weights are not well-aligned with their physiological characteristics. Adjusting closed-loop parameters based on such feedback helps to progressively reduce unnecessary interventions and improve long-term control quality.

### 4.5. Cohort, Architecture, and Stress-Test Analyses

As shown in Table 6,the expanded sensitivity analysis included 30 virtual subjects, each evaluated with three predefined random seeds (90 patient–seed observations). The cohort-average pattern was similar to that of the primary benchmark (TIR, 75.8%; TBR, 4.4%; TBR below 54 mg/dL, 1.1%), while the adolescent subgroup had the highest TBR (5.8%) and worst-k TBR (16.9%). The persistence of the overall pattern across age groups supports robustness within the evaluated conditions. However, the poorer adolescent and worst-k outcomes show that cohort averages do not capture the full physiological heterogeneity. The findings should therefore not be extrapolated directly to the broader T1D population, different treatment behaviors, devices, meal and activity patterns, or clinical settings.

Under a matched protocol, the multi-scale encoder (20/72/288 observations) achieved lower held-out CGM error than the single-scale 20-observation encoder (RMSE, 18.6 versus 20.3 mg/dL; MAE, 12.2 versus 13.5 mg/dL). Descriptive closed-loop outcomes also favored the multi-scale encoder: TIR increased by 3.3 percentage points, whereas TBR and worst-k TBR decreased by 0.9 and 2.8 percentage points, respectively. These results support the utility of multi-scale context under the evaluated protocol, but do not establish clinical benefit.

The warm-start reset was triggered when the CGM rate of change exceeded 3 mg/dL/min for two consecutive cycles or when ensemble disagreement exceeded the training-set 95th percentile. On reset, half of candidates were sampled from a broad diagonal covariance (σ=0.50 U) and half retained the shifted warm-start distribution. Relative to shifted-sequence warm starts alone, this event-triggered reset increased TIR from 65.7% to 68.9%, reduced TBR from 6.2% to 5.1%, reduced worst-k TBR from 18.4% to 15.1%, and shortened recovery time by 26 min, with a 0.03 s increase in planning time per step. This result is bounded by the tested conditions and does not establish global-optimum recovery after arbitrary real-world disturbances.

### 4.6. Unannounced-Meal and Safety-Layer Analyses

The unannounced-meal stress test injected a meal at a fixed evaluation time without supplying meal timing or size to the controller. As shown in Table 7, performance degraded monotonically as meal size increased: TIR fell from 77.6% under nominal conditions to 63.8% after a 90 g unannounced meal, while TBR rose from 3.8% to 6.9% and worst-k TBR from 11.0% to 20.6%. Peak glucose increased to 324 mg/dL and the time to return below 180 mg/dL increased to 196 min at 90 g. The controller therefore inferred meal effects only indirectly from CGM and insulin history. This bounded stress test does not establish robustness to unrestricted real-world meals.

Patient-adaptive safety bounds were determined from insulin sensitivity and prespecified risk strata without using evaluation outcomes, and were applied during both planning rollouts and execution. Compared with uniform bounds, the adaptive mapping increased TIR from 77.6% to 78.4%, reduced TBR from 3.8% to 3.2% and TBR below 54 mg/dL from 1.2% to 0.9%, and reduced the intervention rate from 100% to 72%. The mean adjustment decreased from 1.665 to 0.86 U. These results support physiology-informed personalization but do not establish clinically validated patient-specific safety.

### 4.7. Safety-Oriented Comparator and Data-Source Analyses

Under matched observation streams, subject–seed pairs, action constraints, Safety Layer mapping, and perturbation domains, the proposed MPC achieved TIR of 77.6% and TBR of 3.8%. The corresponding values were 70.2% and 5.6% for PID-AID and 74.9% and 4.8% for the safe-RL comparator. The proposed controller had lower worst-k TBR (7.3%) than PID-AID (12.5%) and safe RL (10.1%) in this implementation-level comparison. These results do not demonstrate equivalence to clinical PID systems or superiority over all safe-RL methods because the optimization procedures and policy classes remain different.

The paired sensitivity analysis distinguished statistical evidence from practical relevance. The proposed controller showed lower TBR than SAC (mean paired difference, −0.101; 95% bootstrap CI, −0.177 to −0.025; Holm-adjusted p=0.048), TD3 (−0.201; −0.278 to −0.124; p<0.001), and PPO (−0.164; −0.207 to −0.121; p<0.001). After Holm adjustment, neither TIR nor TBR differed significantly from safe RL, and the TBR difference from PID-AID was also not statistically significant. These findings represent exploratory comparisons rather than confirmatory evidence of clinical efficacy.

The source-ablation analysis identified measurable separation between MIMIC-IV and Simglucose trajectories (held-out source-classifier AUROC, 0.94; latent-representation MMD, 0.31). Nevertheless, adding MIMIC-IV to Simglucose improved held-out Simglucose RMSE from 19.4 to 18.6 mg/dL and changed closed-loop outcomes from 75.1% TIR and 4.4% TBR to 77.6% and 3.8%, respectively. MIMIC-IV-only training was not eligible for closed-loop evaluation because of interface mismatch. Thus, auxiliary MIMIC-IV data did not degrade measured performance in the target domain, but the source shift remains measurable and outpatient or clinical transfer is not established.

Split-conformal calibration was performed using complete trajectories, with no trajectory contributing time points to more than one split. Nominal empirical coverage was 90.8% for a 90% target (mean interval width, 48.6 mg/dL); under device perturbations, coverage was 89.1% with a 56.3 mg/dL mean interval width; and under unannounced meals, coverage was 87.2% with a 62.4 mg/dL mean interval width. The joint decline in coverage and increase in interval width under stress indicate that the nominal calibration does not fully transfer to the evaluated distribution shifts. These intervals quantify predictive uncertainty but do not establish a closed-loop safety guarantee.

### 4.8. Risk-Objective Ablation

Adding either component of the risk objective improved nominal glycemic outcomes relative to mean-only planning (Table 8). The variance penalty reduced cohort-average TBR from 0.061 to 0.048 and worst-k TBR from 0.184 to 0.146. TailMean alone produced larger reductions, to 0.043 and 0.122, respectively. The complete objective achieved the highest nominal TIR (0.776) and the lowest nominal TBR (0.038), time below 54 mg/dL (0.012), and worst-k TBR (0.110). Relative to mean-only planning, these changes corresponded to a 2.8-percentage-point increase in TIR and reductions of 2.3 percentage points in TBR and 7.4 percentage points in worst-k TBR. CV decreased from 0.381 to 0.358 and total insulin use decreased from 47.8 to 46.2 U/day, whereas runtime increased from 121.0 to 156.3 ms per step.

The same ordering was observed across all perturbation domains (Table 9). Relative to mean-only planning, the complete objective reduced cohort-average TBR from 0.139 to 0.103 under action noise, from 0.102 to 0.068 under CGM noise, from 0.136 to 0.092 under CGM dropout, and from 0.129 to 0.086 under action delay. Worst-k TBR also decreased in every domain, although it remained high under CGM noise (0.305), CGM dropout (0.374), and action delay (0.340). Thus, the complete objective consistently improved the reported tail metrics but did not eliminate hypoglycemia exposure in the most affected virtual-patient subgroup.

### 4.9. Failure-Case Analysis

We conducted a case analysis of four challenging patients who exhibited a Time Below Range (TBR) exceeding 15%. The results, summarized in Table 10, reveal two primary modes of failure. The first is characterized by high sensitivity and high variability; these patients typically display a coefficient of variation (CV) above 0.55 and elevated insulin sensitivity parameters, meaning even small doses can trigger rapid glucose drops that fall outside the predictive scope of the current planning model. The second mode involves planning lag and insufficient regulation; in scenarios of high volatility, the speed of plan updates and the intensity of safety corrections fail to keep pace with sudden changes, leading to an accumulation of hypoglycemia risk.

These failure cases demonstrate that the current framework still suffers from predictive and regulatory lag when dealing with extremely sensitive individuals. To address this, future improvements could focus on three areas: first, introducing more granular modeling of the postprandial phase to enhance the detection of rapid, short-term glucose drops; second, implementing adaptive safety boundaries for highly sensitive patients to trigger protective dose reductions proactively; and third, incorporating the trajectories of these failure cases into retraining protocols to improve the model’s ability to handle extreme states.

It is important to emphasize that this analysis of failure cases does not invalidate the primary conclusions; rather, it serves to clarify the system’s operational boundaries and establish priorities for improvement. By identifying the mechanisms behind these failures, future optimizations can target “tail-end” safety more effectively, avoiding the pitfall of blindly increasing conservatism across the entire patient population.

### 4.10. Worst-k and Tail Risk Analysis

In clinical decision-making, high-risk, susceptible patients are a primary focus; to investigate the system’s dynamic resilience against such risks, this section experimentally analyzes the control performance for these patients. As shown in Table 11, the Time Below Range (TBR) for susceptible patients in the tail (worst-case) scenario is significantly higher than the average, typically ranging from two to four times the mean value. While the average TBR is 3.8% (approximately 55 min of hypoglycemia per day)—meeting the clinical standard of staying below 4%—the TBR surges to 11% (approximately 2.6 h of hypoglycemia per day) in the worst-k scenario, far exceeding the clinical threshold. This disparity between tail risk and the average level reaches 189%, representing a 2.9-fold increase.

The study further reveals that the magnitude of this tail-risk amplification varies depending on the type of disturbance. As shown in Table 12, specific disturbance domains drastically exacerbate risk; notably, in the CGM signal loss domain, the TBR for the worst-k scenario skyrockets to 37.4%—a 240% increase over the 11% observed in the nominal domain. Action delays and CGM noise follow closely, with worst-k TBR values of 34.0% and 30.5%, respectively.

An analysis of the underlying mechanisms indicates that sensor signal loss directly impairs state estimation capabilities. For highly sensitive patients whose blood glucose fluctuations are difficult to predict, such information loss is critical, vastly increasing the likelihood of system failure. It is worth noting that while the decline in Time In Range (TIR) at the tail end remains relatively consistent across different domains (ranging from 31% to 38%), fluctuations in TBR are far more pronounced, demonstrating that TBR is a more sensitive metric for assessing tail risk.

## 5. Discussion

The central finding was a divergence between cohort-average control and tail risk. The primary unguided iCEM-MPC controller met nominal reference targets for average TIR and TBR, yet nominal worst-k TBR was 11.0%. Under CGM dropout, worst-k TBR reached 37.4% despite a cohort-average TBR of 9.2%. Average target attainment therefore did not imply uniformly acceptable control across patients.

The four-way risk-objective ablation clarified which objective terms were associated with this pattern. Variance penalization and TailMean each improved nominal TBR and worst-k TBR relative to mean-only planning. TailMean produced the larger descriptive reduction, and the complete risk objective yielded the lowest values across all five domains. However, these ablations lacked uncertainty intervals and paired inferential analyses. They therefore support the combined objective as the best observed configuration but do not resolve independent component effects or their interaction.

The perturbation results further showed why cohort and tail summaries should be reported together. Across the four stress tests, cohort-average TIR changed by no more than 5.3 percentage points from nominal. In contrast, worst-k TBR ranged from 24.0% to 37.4%. CGM dropout produced the highest absolute tail risk, which is consistent with impaired state estimation when observations are missing. This interpretation remains provisional because the study did not isolate prediction error as the causal mechanism.

The Safety Layer modified every proposed action in the analyzed run, with a mean adjustment of 1.665 insulin units per control step. This intervention pattern indicates a systematic mismatch between planner proposals and the programmed feasible set. Projection enforced the action bounds, but neither projection nor the high intervention rate ensured safe future glucose trajectories.

Comparisons with PPO, SAC, and TD3 favored the primary controller on the reported metrics, but they should be interpreted at the implementation level. Sharing the same Safety Layer reduced one source of imbalance without matching objectives, policy classes, or optimization procedures. Similarly, the optional RL-guided proposal variant changed tail outcomes under a fixed sampling budget, but the planners were not compared at matched convergence. The available evidence therefore supports an exploratory search-efficiency interpretation rather than an intrinsic safety benefit from RL guidance.

Several limitations define the scope of these findings. The primary evaluation used 10 virtual patients and three random seeds, while a separate sensitivity analysis included 30 virtual patients across adult, adolescent, and child groups. The primary cohort was a prespecified reproducible benchmark subset with complete 24 h meal schedules and no missing model parameters; neither cohort represents the clinical T1D population. The results may not generalize to different treatment behaviors, devices, meal and activity patterns, or clinical settings. Clinical validation was not performed. MIMIC-IV served only as auxiliary representation-learning data and differs from outpatient T1D management. Ensemble disagreement itself was not calibrated, although separate conformal intervals were assessed. Multi-step world-model accuracy was not independently evaluated. The baseline tests were exploratory, and the risk-objective ablation lacked uncertainty estimates. Time below 54 mg/dL was available for the nominal risk-objective ablation but not consistently across methods and conditions. Finally, predefined perturbations and common action bounds cannot represent the full diversity of patients, sensors, pumps, or failure processes.

Taken together, the framework is best viewed as a preclinical method for exposing and optimizing tail risk rather than as a deployable insulin controller. Further validation should prioritize patient-specific action bounds, calibrated predictive uncertainty, explicit missing-CGM fallback behavior, and matched-budget planner comparisons.

## 6. Conclusions

This study developed a tail-risk-aware iCEM-MPC framework that combines latent-state prediction, risk-sensitive planning, and auditable action projection. Under the evaluated conditions, the complete risk objective improved average and worst-k outcomes relative to mean-only planning, while the primary controller met nominal cohort-average glycemic targets. These gains did not eliminate tail risk: worst-k TBR remained elevated under sensing and actuation perturbations, and the Safety Layer modified every proposed action in the analyzed run. The framework therefore enables average performance, vulnerable-patient outcomes, and dependence on action constraints to be evaluated together. Clinical safety and efficacy remain unestablished, and patient-specific safeguards require future validation beyond the primary uniform-bound configuration.

## Figures and Tables

**Table 1:** MDP formulation of closed-loop insulin delivery.

Component	Symbol	Definition
State space	S	$\left\{g\in \left[50,400\right],\;i\in R_{\geq 0},\;m\in R_{\geq 0}\right\}$
Action space	A	$a_{t}\in \left[0,a_{\max}\right]$, $a_{\max}=10$ units/step
Transition dynamics	$p(s_{t+1}|s_{t},a_{t})$	UVA/Padova T1D simulator
Reward function	$r(s_{t},a_{t})$	$w_{1}I\left[g_{t}\in \left[70,180\right]\right]-w_{2}I\left[g_{t}<70\right]-w_{3}I\left[g_{t}>250\right]-ca_{t}$
Discount factor	γ	γ∈(0,1)
Policy	$\pi (a_{t}|z_{t})$	Stochastic insulin dosing distribution
Value function	$V^{\pi }\left(z\right)$	$E_{\pi }\;\left[\sum_{t}\gamma^{t}r\left(s_{t},a_{t}\right)|s_{0}=z\right]$
Action-value function	$Q^{\pi }\left(z,a\right)$	$E_{\pi }\;\left[\sum_{t}\gamma^{t}r\left(s_{t},a_{t}\right)|s_{0}=z,a_{0}=a\right]$

**Table 2:** Definitions of evaluation metrics.

Metric	Definition	Unit	Clinical Threshold
TIR	Time in target range 70–180 mg/dL	proportion	≥70% (ADA goal)
TBR	Time below range < 70 mg/dL	proportion	<4% (alert threshold)
CV	Coefficient of variation (std/mean)	scalar	<0.36 (stability threshold)
Worst-k TBR	Mean TBR among the 20% of virtual patients with the highest TBR	proportion	not established
Intervention intensity $I_{1}$	Mean L1 adjustment per step	insulin units	—
Intervention rate	Fraction of steps with adjustment	proportion	—

**Table 3:** Data composition for auxiliary training.

Source	Transitions	Episodes	Description
Simglucose	46,353	225	Virtual patient, closed-loop control
MIMIC-IV	37,247	1000	Auxiliary ICU glucose–insulin records
Total	83,600	1225	Combined representation-learning data

**Table 4:** Experimental configuration for the primary and sensitivity analyses.

Component	Setting
History windows; Transformer layers; latent dimension; heads	20/72/288 observations; L=3; d=128; 4 heads
Feed-forward dimension; dropout; ensemble size	512; 0.10; M=5
Training	AdamW ($10^{-4}$ weight decay), $3\times 10^{-4}$ cosine schedule, 5-epoch warm-up; batch size 256
Training stopping rule	150 epochs maximum; early stopping after 15 validation-RMSE epochs
MPC/iCEM	H=12, N=64, 8 iterations, elite fraction 10%, η=0.20
Risk rollouts	12 perturbation rollouts/candidate; tail fraction ρ=0.20
Safety bounds	$0\leq a_{t}\leq 3.0$ U and $|a_{t}-a_{t-1}|\leq 0.75$ U

**Table 5:** Exploratory paired statistical summaries for the evaluated outputs.

Method	Metric	Mean Diff	95% CI	Paired $d_{z}$	Holm-Adjusted *p*
PPO	TIR	+0.448	[0.391, 0.505]	2.86	<0.001
PPO	TBR	−0.164	[−0.207, −0.121]	−1.37	<0.001
SAC	TIR	+0.017	[−0.056, 0.090]	0.08	0.999
SAC	TBR	−0.101	[−0.177, −0.025]	−0.48	0.048
TD3	TIR	+0.105	[0.029, 0.181]	0.50	0.042
TD3	TBR	−0.201	[−0.278, −0.124]	−0.95	<0.001

**Table 6:** Expanded virtual-cohort sensitivity analysis.

Analysis Set	Subjects	TIR	TBR	TBR < 54	Worst-k TBR
Primary benchmark	10	77.6%	3.8%	1.2%	11.0%
Expanded cohort	30	75.8%	4.4%	1.1%	13.2%
Adults	12	77.9%	3.7%	0.8%	10.4%
Adolescents	10	72.6%	5.8%	1.8%	16.9%
Children	8	76.1%	3.9%	1.0%	12.7%

**Table 7:** Unannounced-meal stress test.

Scenario	TIR	TBR	TBR < 54	Worst-k TBR	Peak Glucose
Nominal	77.6%	3.8%	1.2%	11.0%	221
Unannounced 30 g	74.1%	4.3%	1.3%	12.4%	248
Unannounced 60 g	69.0%	5.2%	1.7%	15.8%	286
Unannounced 90 g	63.8%	6.9%	2.4%	20.6%	324

**Table 8:** Descriptive risk-objective ablation under nominal conditions. TBR54 denotes time below 54 mg/dL. No uncertainty intervals or hypothesis tests were available for this ablation.

Objective	TIR	TBR	TAR	TBR54	Worst-k TBR	CV	Insulin (U/Day)	Runtime (ms/Step)
Mean only	0.748	0.061	0.191	0.024	0.184	0.381	47.8	121.0
Mean + variance	0.762	0.048	0.190	0.018	0.146	0.371	47.1	136.2
Mean + TailMean	0.769	0.043	0.188	0.014	0.122	0.364	46.6	145.4
Complete objective	0.776	0.038	0.186	0.012	0.110	0.358	46.2	156.3

**Table 9:** Descriptive risk-objective ablation across operating conditions. Each entry reports cohort-average TBR/worst-k TBR.

Objective	Nominal	Action Noise	CGM Noise	CGM Dropout	Action Delay
Mean only	0.061/0.184	0.139/0.318	0.102/0.382	0.136/0.451	0.129/0.419
Mean + variance	0.048/0.146	0.121/0.276	0.084/0.343	0.111/0.407	0.105/0.371
Mean + TailMean	0.043/0.122	0.112/0.254	0.076/0.322	0.101/0.391	0.095/0.357
Complete objective	0.038/0.110	0.103/0.240	0.068/0.305	0.092/0.374	0.086/0.340

**Table 10:** Glycemic outcomes for four high-TBR virtual-patient cases.

Patient	TIR	TBR	CV	Hypoglycemia Minutes
P1	0.487	0.389	0.570	560
P2	0.556	0.274	0.552	395
P3	0.650	0.261	0.443	376
P4	0.685	0.169	0.423	243

**Table 11:** Cohort-average and worst-k outcomes across domains.

Domain	TIR	Worst-k TIR	Diff	TBR	Worst-k TBR	Diff
Nominal	0.776	0.502	35%	0.038	0.110	189%
Action noise	0.726	0.481	34%	0.103	0.240	133%
CGM noise	0.776	0.523	33%	0.068	0.305	349%
CGM dropout	0.723	0.450	38%	0.092	0.374	307%
Action delay	0.727	0.501	31%	0.086	0.340	295%

**Table 12:** Detailed analysis of different domains.

Domain	Worst-k TBR	Diff of Nominal
Nominal	0.110	baseline
Action noise	0.249	118%
CGM noise	0.305	177%
CGM dropout	0.374	240%
Action delay	0.340	209%

## Data Availability

No new clinical data were created in this study. This study reused MIMIC-IV data, which are available through PhysioNet subject to the database access requirements. The processed evaluation data and code implementing the model are available from the corresponding authors upon reasonable request.
